# Dealing With Human Resources in the Age of Consumer 4.0: Aiming to Improve Service Delivery

**DOI:** 10.3389/fpsyg.2019.03058

**Published:** 2020-01-21

**Authors:** Juan Jose Blazquez-Resino, Santiago Gutiérrez-Broncano, Pablo Ruiz-Palomino, Pedro Jimenez-Estevez

**Affiliations:** ^1^Department of Business Administration, University of Castilla La Mancha, Talavera de la Reina, Spain; ^2^Department of Business Administration, University of Castilla La Mancha, Cuenca, Spain; ^3^Department of Business Administration, University of Castilla La Mancha, Toledo, Spain

**Keywords:** Industry 4.0, Marketing 4.0, Customer 4.0, human resources, service, I.O.T, business ecosystems, skills and competences 4.0

## Introduction

The evolution of information and communication technologies (ICTs) has produced seismic, global shifts in recent decades. The development of ICTs, coupled with the progressive digitization processes they drive, has helped to create new opportunities for growth—as well as new challenges for decision-making. For instance, companies in today's environment must contend with, among other things, large databases and the need to increase the productivity of different tasks and operations. At the same time, ICTs allow new business models wherein consumers can become active co-producers in the value creation process (Dellaert, 2019). However, in order to shift innovation from the company to the customer, firms need to achieve a different kind of coordination between various organizational areas, including marketing (Ardito et al., 2019).

In this sense, firms face several hurdles, such as training employees to handle more strategic, cooperative, and creative tasks that entail greater responsibilities. Employees must not only acquire the necessary skills to participate in virtual work; they must also be able to engage the co-production and value generation processes with consumers. On this basis, this opinion piece aims to highlight some changes that companies should implement in order to properly train, qualify, and manage employees in the current context of Industry 4.0—especially if they want to meet the needs of today's consumers.

## Changes in Consumer Behavior in the Modern Era

Consumer behavior is currently characterized by the growing demand for technology. Customers are hyper-connected through different technologies, including not only the well-known mobile technologies, but also those like Internet of Thing (IOT), nanotechnology, or artificial intelligence. These technologies underlie several defining activities of Consumer 4.0, such as the co-design and recommendation of products; participating in the distribution and sale of products; co-producing and sharing experiences; and offering peer support, revision, and innovation (Dellaert, 2019). Consumers also have the opportunity to access a huge amount of information, which allows them to compare offers from a wide variety of suppliers. Consumers not only look for products that meet their needs; they also want to be part of the product through active participation in co-production and value generation. Thus, consumers need to feel tethered to the product by participating in its creation, interacting with it and sharing their experiences (Martínez-Cañas et al., 2016).

The notion of meeting consumer needs through co-production (Wu and Liu, 2018) is indispensable in the current context of Marketing 4.0. This new approach of Marketing combines online and offline interactions between customers and businesses, to achieve a better way to serve their products and generate value, establishing more flexible and adaptive processes. Therefore, companies should not simply transmit their own values and contributions; they should also develop a deep co-construction process, based on offline and online interactions and partnerships with customers and followers. In this vein, several studies from the last decade (e.g., Payne et al., 2008; Edvardsson et al., 2011; Martínez-Cañas et al., 2016) have highlighted the need for new theories to better understand consumer co-creation processes, especially in coordination with ICTs. After all, consumers can provide online communities with more valuable information than producers by sharing their experiences through social networks (Kotler et al., 2016).

In order to remain competitive, companies must undergo a digital transformation that affects not only the areas of value, but also the ways in which they interact with their operational environment. The following section will discuss the implications of this transformation for human resource training.

## Implications for Human Resources Formation and Management

An organization's performance and competitiveness depend largely on how its employees are trained and managed. Developing and maintaining a highly committed and qualified workforce means investing in proper training and human resource management processes (Hecklau et al., 2016). Beyond the selection, hiring and dismissal of employees, the management of human resources must be focused on employees' development through education and training. Despite the importance of human resource management in achieving a company's objectives, there are few scientific papers that try to generate knowledge on developing human resource strategies within Industry 4.0 (Hecklau et al., 2016; Benešová and Tupa, 2017; Longo et al., 2017). This new context routinely blurs the lines between personal and professional activities, as well as the forms of employing people who participate in value creation systems (Dellaert, 2019).

This “fourth industrial revolution” is characterized by a paradigm shift oriented toward the decentralization of production processes, facilitating the integration of different company functionalities and members of the distribution channel. As Schrauf and Berttram (2016, p. 4) stated, “the chain becomes a completely integrated ecosystem that is fully transparent to all the players involved.” The ultimate goal must be to meet customized market demands in a short period of time while using the least amount of energy and resources. Companies must be closer to their customers and more reactive when interpreting their needs, which they can achieve by involving customers in the design and development of product processes (Bettiol et al., 2017). As a result, and parallel with previous industrial revolutions, some professions and jobs may disappear (Benešová and Tupa, 2017) in the wake of improved procedural efficiency and employee training.

In this regard, it should be clarified that Industry 4.0 is a new phenomenon aimed at changing the economic rules in all industries, albeit with special attention to manufacturing (c.f., Kagermann et al., 2013; Weyer et al., 2015; Stock and Seliger, 2016). The peculiar feature of this industrial revolution is its greater degree of complexity compared to previous ones. The underlying idea of Industry 4.0 leaves aside the traditional paradigms of production and management. Instead, it considers the digitization of the company and the entire value chain, based on the adoption of digital technologies that can help companies establish real-time connections between people, objects and systems (Hecklau et al., 2016). The relevant literature has revealed interesting findings about the beneficial effects of Industry 4.0, such as companies' increasing productivity and customers' growing role in productive processes (Bettiol et al., 2017).

In this framework, the so-called Smart Factories of the future will allow employees to work alongside customers and suppliers in a digital ecosystem in ways that improve value chain management and marketing functions (Ardito et al., 2019). In this sense, the concept of Industry 4.0 is related to other technological terms such as radio frequency identification (RFID), Internet of Things (IoT), cloud computing, decision-making/supporting systems, computational intelligence, and data mining (Wang et al., 2017), which can be used to bolster the company's knowledge of customer needs (Porter and Heppelmann, 2015).

In short, the growing implementation of ICT entails a need to significantly improve employees' training and qualification with regard to new technologies and smart media. Benešová and Tupa (2017) noted that the development of technologies has an important impact on education systems, where only qualified and highly trained employees will be able to control these technological tools. In order to meet consumers' needs in terms of functionality, quality and service, Industry 4.0 may need to be accompanied by an Education 4.0 system that combines both real and virtual information (Benešová and Tupa, 2017). Indeed, Industry 4.0 involves tasks of greater complexity that require human operators with more experience or adaptive capabilities (Longo et al., 2017).

## Discussion

This opinion article has primarily highlighted, among other issues, the need to establish a coordination between Industry 4.0 and Marketing 4.0, based on the study of the processes of educating, training and management of human resources to offer better performance to Consumer 4.0. Undoubtedly, Industry 4.0 is helping to create many new opportunities for companies; nonetheless, increasing automation and digitization introduce several critical challenges. Faced with an increasing amount of virtual and flexible work topics, employees need to adopt a higher volume of responsibilities that involve strategic, cooperative, and creative thinking. To this end, human resource management processes must be geared toward training employees to not only use new technologies, but also understand and adapt to customers' new role in the process. Therefore, it is important to focus attention on human resource management in relation to the latest marketing approaches inherent to Marketing 4.0. The need to strengthen the union of human resources management from a marketing perspective, established through Internal Marketing, is not surprising.

Obviously, industry has undergone several revolutions that ultimately changed not only production, but also the labor market and education system. In this latest iteration, employees need training on how to develop their activities in relation to their direct and continuous contact with customers. Going forward, customers' perceptions of products—their quality, variety, and delivery—will be strongly influenced by information technologies and social media networks. In this context, companies should try to be flexible and quickly adapt their structures, products and brands to changing social trends. Production processes must be efficient enough to meet customers' needs, but also flexible enough to adapt swiftly to changes in customer demand. In this regard, the ability to perform timely analysis of market data is a key element of flexible production. Thus, companies need to invest in communication channels that facilitate the continuous exchange of information on individual needs and situations in real time.

In sum, companies must strive to understand their customers more intimately and involve them in the design and development of products. That said, co-production is a great challenge for human resource training and management. Employees need to acquire the skills necessary for virtual work and be cognizant of the blurring lines between personal and professional activities in value creation systems.

## Author Contributions

All authors listed have made a substantial, direct and intellectual contribution to the work, and approved it for publication.

### Conflict of Interest

The authors declare that the research was conducted in the absence of any commercial or financial relationships that could be construed as a potential conflict of interest.
